# Genetic Characterization of the Ibuprofen-Degradative Pathway of Rhizorhabdus wittichii MPO218

**DOI:** 10.1128/aem.00388-22

**Published:** 2022-05-23

**Authors:** Magaly Aulestia, Amando Flores, Sebastián Acosta-Jurado, Eduardo Santero, Eva M. Camacho

**Affiliations:** a Centro Andaluz de Biología del Desarrollo/CSIC/Universidad Pablo de Olavide/Junta de Andalucía, Departamento de Biología Molecular e Ingeniería Bioquímica, Seville, Spain; University of Tartu

**Keywords:** biodegradation pathway, biodegradative plasmid, emerging contaminant, genetics, ibuprofen, *R. wittichii* MPO218

## Abstract

Ibuprofen is one of the most common drugs found as a contaminant in soils, sediments, and waters. Although several microorganisms able to metabolize ibuprofen have been described, the metabolic pathways and factors limiting biodegradation in nature remain poorly characterized. Among the bacteria able to grow on ibuprofen, three different strains belonging to *Sphingomonadaceae* and isolated from different geographical locations carry the same set of genes required for the upper part of the ibuprofen metabolic pathway. Here, we have studied the metabolic pathway of Rhizorhabdus wittichii MPO218, identifying new genes required for the lower part of the ibuprofen metabolic pathway. We have identified two new DNA regions in MPO218 involved in the metabolism of ibuprofen. One is located on the MPO218 chromosome and appears to be required for the metabolism of propionyl-CoA through the methylmalonyl-CoA pathway. Although involved in ibuprofen metabolism, this region is not strictly necessary for growing using ibuprofen. The second region belongs to the pIBU218 plasmid and comprises two gene clusters containing aromatic compound biodegradation genes, part of which are necessary for ibuprofen degradation. We have identified two genes required for the first two steps of the lower part of the ibuprofen metabolic pathway (*ipfL* and *ipfM*), and, based on our results, we propose the putative complete pathway for ibuprofen metabolism in strain MPO218.

**IMPORTANCE** Ibuprofen, one of the most common pharmaceutical contaminants in natural environments, is toxic for some aquatic and terrestrial organisms. The main source of environmental ibuprofen is wastewater, so improving wastewater treatment is of relevant importance. Although several microorganisms capable of biodegrading ibuprofen have been described, the metabolic pathways and their genetic bases remain poorly understood. Three bacterial strains of the family *Sphingomonadaceae* capable of using ibuprofen as carbon and energy source have been described. Although the genes involved in the upper part of the degradation pathway (*ipfABDEF* cluster) have been identified, those required for the lower part of the pathway remained unknown. Here, we have confirmed the requirement of the *ipf* cluster for the generation of isobutyl catechol and have identified the genes involved in the subsequent transformation of the metabolic products. Identification of genes involved in ibuprofen degradation is essential to developing improved strains for the removal of this contaminant.

## INTRODUCTION

Analgesics and nonsteroidal anti-inflammatory drugs (NSAIDs), such as ibuprofen (IBU), naproxen, diclofenac, etc., are the pharmaceutical compounds that appear most frequently as contaminants in soils, sediments, and waters (1–4). Although pharmaceutical pollutants can be the result of inappropriate disposal of unused drugs, waste from the pharmaceutical industry, etc., the main source of pharmaceuticals in the environment is drugs discharged into wastewater. Part of the pharmaceutical compounds used as treatments are not completely metabolized and are afterward excreted in the feces and urine as unchanged compounds or as metabolites (3, 5–7).

Incomplete removal during wastewater treatment reported for conventional wastewater treatment plants results in the presence of NSAIDs in discharged effluent or adsorbed into activated sludge at concentrations up to μg L^−1^. In addition to effluent discharge, the use of sewage sludge as fertilizer and irrigation with reclaimed water introduce these pharmaceutical products into agricultural soil from where they can leach into groundwater. Thus, pharmaceutical compounds have been detected as contaminants in widespread terrestrial and aquatic environments (1, 3–5, 7–9).

IBU is one of the most widely used NSAIDs in the world and is on the World Health Organization's list of essential medicines (10). Up to 15% of the ingested IBU is excreted unchanged or as metabolites, and IBU has been detected in various environmental samples, including wastewater, sludge, soils, surface water, groundwater, and seawater, at concentrations ranging from ng L^−1^ up to 300 μg L^−1^ (2, 7, 11). Several studies have shown a toxic effect on aquatic and soil organisms exposed to IBU concentrations equivalent to those found in the environment (2, 12–14), so improving wastewater treatment processes to eliminate this pollutant is a priority. Although several microorganisms capable of biodegrading IBU have been isolated to date, the metabolic pathways of this biodegradation and their genetic bases remain poorly understood (2, 15–17). Although ibuprofen seems to be biodegradable, the fact that it is detected in such large amounts in the environment, and that it is detected in wastewater treatment plant (WWTP) effluents, indicates that there must be environmental factors limiting its biodegradation (2, 7, 11, 18). Knowing the IBU biodegradation pathways, as well as their regulation, could allow the development of more efficient bioremediation strategies.

Within the *Sphingomonadaceae* family, three different strains sharing the same set of genes related to IBU biodegradation have been independently isolated worldwide. The *ipf* cluster, first described in the *Sphingomonas* ibu-2 strain by Murdoch et al. (15, 19), has been found highly conserved at the DNA level in two different strains, Rhizorhabdus wittichii MPO218 (16) (formerly Sphingomonas wittichii [20]) and Sphingopyxis granuli RW412 (17). Both MPO218 and RW412 carry the *ipf* genes on a large plasmid (16, 17) that, in the case of the plasmid pIBU218 carried by R. wittichii strain MPO218, has been shown to be conjugative (16).

Despite having the same set of *ipf* genes, which, according to Murdoch et al. (15, 19), code for the upper part of the IBU metabolic pathway, the three strains grow differently using IBU as a carbon and energy source. Thus, while Ibu-2 accumulates a yellow intermediate while growing with IBU as the sole carbon and energy source, neither RW412 nor MPO218 develop any coloration under the same conditions. In addition, the doubling time of MPO218 growing on IBU was substantially shorter than that of RW412, around 3 h for MPO218 versus the 16 h described for RW412 (16, 17). Murdoch et al. (15, 19) and Aguilar-Romero et al. (17) have analyzed the metabolic pathway of ibuprofen biodegradation at the biochemical level showing that, in the first step, a CoA ligase binds coenzyme A to ibuprofen, which is then attacked by a dioxygenase system to form a dihydrodiol, which thereafter is cleaved by a retro-aldolase into propionyl-CoA and 4-isobutylcatechol. According to gas chromatography-mass spectrometry (GC-MS) metabolite analysis by Murdoch et al. (19), 4-isobutylcatechol should subsequently be attacked by an extradiol-2,3 dioxygenase and a dehydrogenase to produce 2-hydroxy-5-isobutylhexa-2,4-dienoic acid. Although genes encoding these activities are located in the regions flanking the *ipf* cluster, the genes involved in the lower pathway had not been determined so far.

Using a classical genetic approach, in this work we have studied the metabolic pathway of R. wittichii MPO218, identifying the genes required for IBU metabolism in this strain. In addition to the *ipf* region described above, we have identified two other DNA regions involved in IBU utilization. One of the regions, involved but not essential for growth using IBU, is involved in the metabolism of propionyl-CoA, supporting the proposal that propionyl-CoA is a by-product of IBU degradation (15, 17, 19). The other region is also found on pIBU218 but is far from the *ipf* region and is essential for allowing growth using IBU. This region is required for the metabolism of 4-isobutylcatechol, the product of the upper part of the IBU biodegradation pathway (15, 17, 19). Based on our results, we propose the putative complete pathway for IBU catabolism in MPO218.

## RESULTS

### Identification of IBU genes.

To identify the genes necessary to grow on IBU as a carbon and energy source, we performed transposon mutagenesis with miniTn*5*-Km and selected Ibu-negative (Ibu^−^) mutants unable to use IBU as a nutrient but able to grow in minimal medium with β-hydroxybutyrate (BHB), a different carbon source. Three different phenotypes were detected among the mutants with impaired growth on IBU, including (i) mutants unable to grow that do not modify the growth medium, (ii) mutants unable to grow that produce dark brown coloration of the growth medium in the presence of IBU, and (iii) a leaky mutant able to poorly grow on IBU (Table 1; see also Fig. S1 at https://rio.upo.es/xmlui/handle/10433/12901).

**TABLE 1:** Descriptions of Ibu^−^ mutants^*a*^

Phenotype	Mutant	Genomic localization	Locus	Functional annotation	Deletion
Colorless Ibu^−^	MIBU8	pIBU218	MPO218_05532	Glutathione *S*-transferase	ND
	MIBU12	pIBU218	MPO218_05528	Thiolase/acyl transferase	ND
	MIBU11	Chr	MPO218_01910	Hypothetical protein	Region III
	MIBU20	Chr	MPO218_00237	Cell division protein FtsQ	Region III
	MIBU24	Chr	MPO218_03925	Aconitate hydratase	Region III
	MIBU29	pIBU218	MPO218_05532	Glutathione *S*-transferase	ND
Leaky	MIBU18	Chr	MPO218_03758	Propionyl-CoA carboxylase beta-chain	ND
dark Ibu^−^	MIBU1	Chr	MPO218_05027	TonB-dependent receptor	Region I
	MIBU3	Chr	MPO218_00050	Hypothetical protein	Region I
	MIBU7	pIBU218	MPO218_05607	Integrase-like protein	Region I
	MIBU13	pIBU218	MPO218_05469	5-Carboxymethyl-2-hydroxymuconic-semialdehyde dehydrogenase	ND
	MIBU23	pIBU218	MPO218_05652	Catechol 2,3-dioxygenase	ND

a “Genomic localization” indicates if insertions were located on the MPO218 chromosome (Chr) or on plasmid pIBU218. “Deletion” indicates if mutants present deletion that affects region I or region III of plasmid pIBU218 (see Fig. 1). ND, not determined.

To identify the genes affected by the transposons, we sequenced the DNA regions flanking the insertions. The results indicated that mutations were located in three main regions (Table 1) as follows: (i) the noncolored mutants are affected in the *ipf* region previously described as necessary for IBU metabolism (15, 16), which is located in pIBU218 (region III in Fig. 1); (ii) the dark mutants are affected in a region containing genes related to biodegradation of aromatic compounds and that is also located on pIBU218 (16) (region I in Fig. 1); and (iii) the leaky mutant is affected in a gene annotated as a beta subunit of a propionyl-CoA carboxylase carboxyl transferase, located on the MPO218 chromosome. Interestingly, we also found that a few mutants, either brown or noncolored, had insertions located in different regions of the MPO218 genome and were apparently not related to aromatic compound utilization (see below).

**FIG 1:**
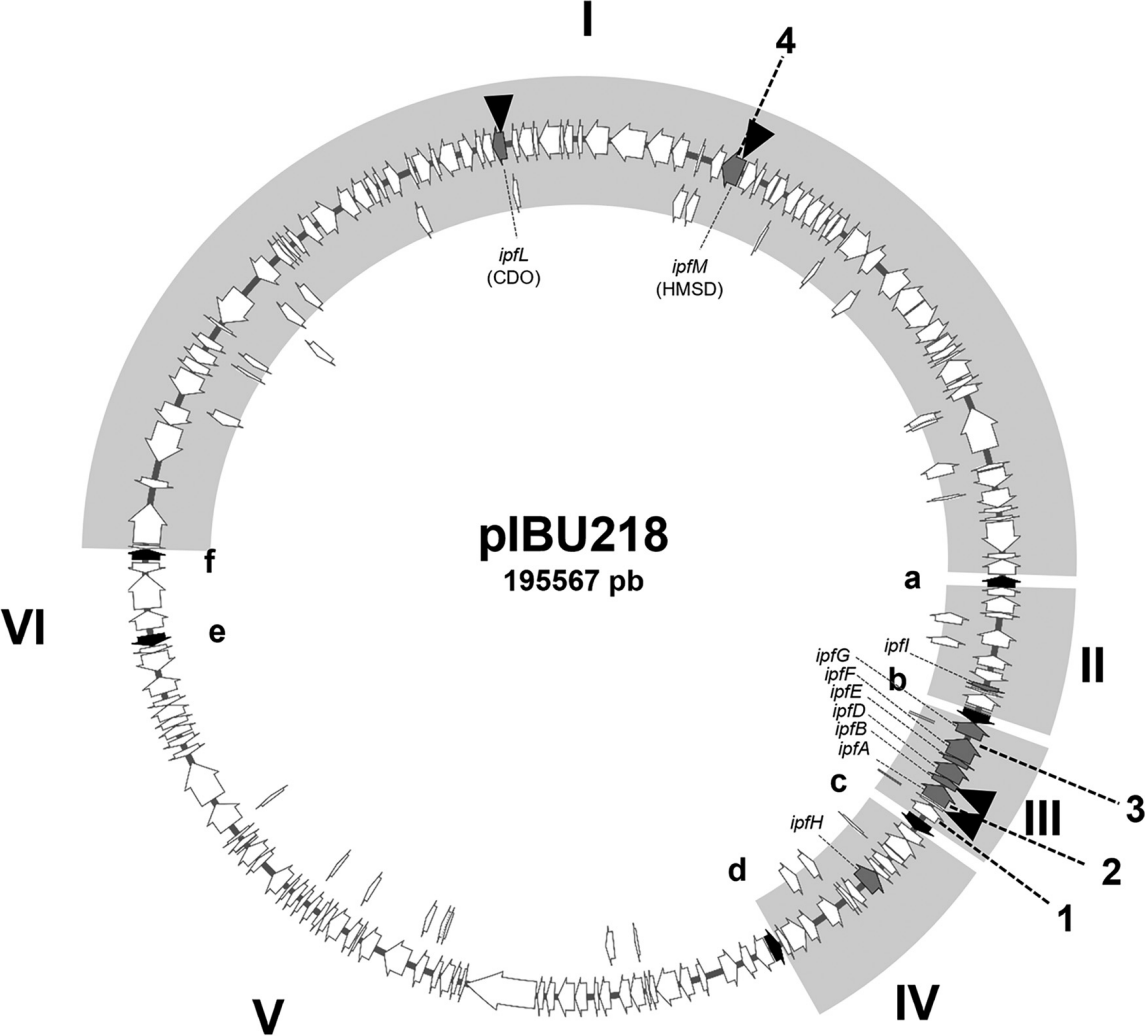
Schematic representation of plasmid pIBU218. Gray arrows indicate genes implicated on ibuprofen biodegradation; black arrows, designated a to f, represent IS*6100* elements. Regions between IS*6100* elements are named I to VI, and those regions previously demonstrated as necessary for ibuprofen metabolism are indicated as gray boxes (16). Black triangles indicate the position of miniTn*5* insertions. Numbers 1 to 4 indicate the DNA sequences analyzed by PCR amplification.

### Characterization of the noncolored Ibu^−^ mutants.

As explained above, noncolored mutants were classified into two types, (i) mutants containing miniTn*5* insertions in the *ipf* region of pIBU218 (MIBU8, MIBU12, and MIBU29), and (ii) mutants containing insertions in other unrelated DNA regions (MIBU11, MIBU20, and MIBU24) (Table 1). Considering the reported instability of pIBU218 (16), we speculated that the phenotype of the latter type of mutants could be due to deletions of plasmid regions and not to the miniTn*5* insertion located elsewhere. To evaluate this possibility, we analyzed the integrity of the *ipf* region by PCR amplification using primers to amplify *ipfA* (number 2 in Fig. 1). As shown in Table 1, the three noncolored mutants unable to grow using IBU that contain the transposon at sites other than the *ipf* region (MIBU11, MIBU20, and MIBU24) did not amplify the *ipfA* gene, indicating that the *ipfA* gene was deleted in these mutants. In order to test the possible deletion of the whole cluster, we checked the presence of other *ipf* genes by PCR amplification. As indicated in Table 1, the results confirmed that MIBU11, MIBU20, and MIBU24 have a deletion that includes at least the entire central *ipf* cluster flanked by the IS*6100* elements (region III, numbers 1, 2, and 3 in Fig. 1).

**FIG 2:**
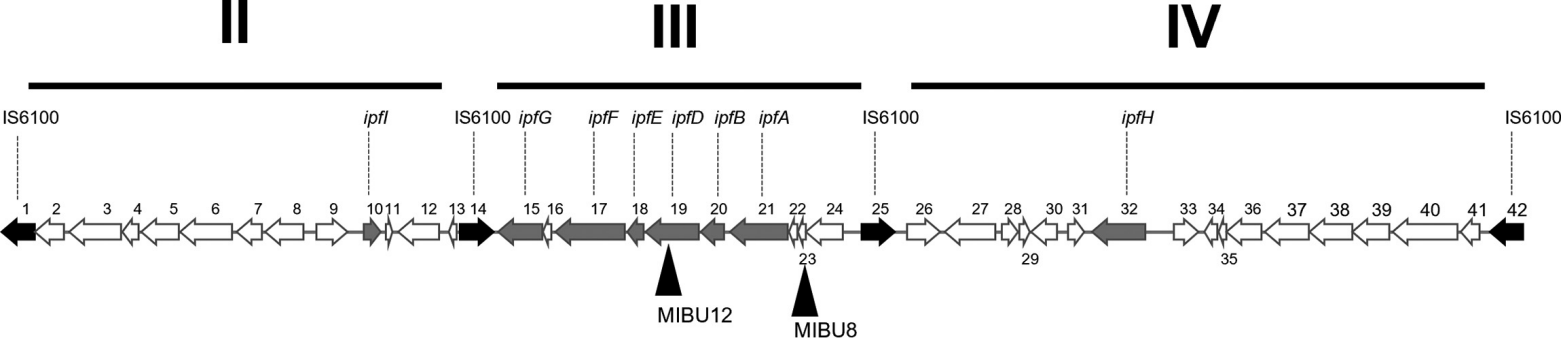
Schematic representation of the *ipf* region of plasmid pIBU218. Figure represents the pIBU218 regions II, III, and IV. Arrows represent coding DNA sequences (CDS) from MPO_005510 (number 1) to MPO_00551 (number 42) (for annotation details, see Table S1 at https://rio.upo.es/xmlui/handle/10433/12901). Gray arrows indicate *ipf* genes, and black arrows show IS*6100* elements. Black triangles indicate the position of miniTn*5* in mutants MIBU8 and MIBU12.

**FIG 3:**
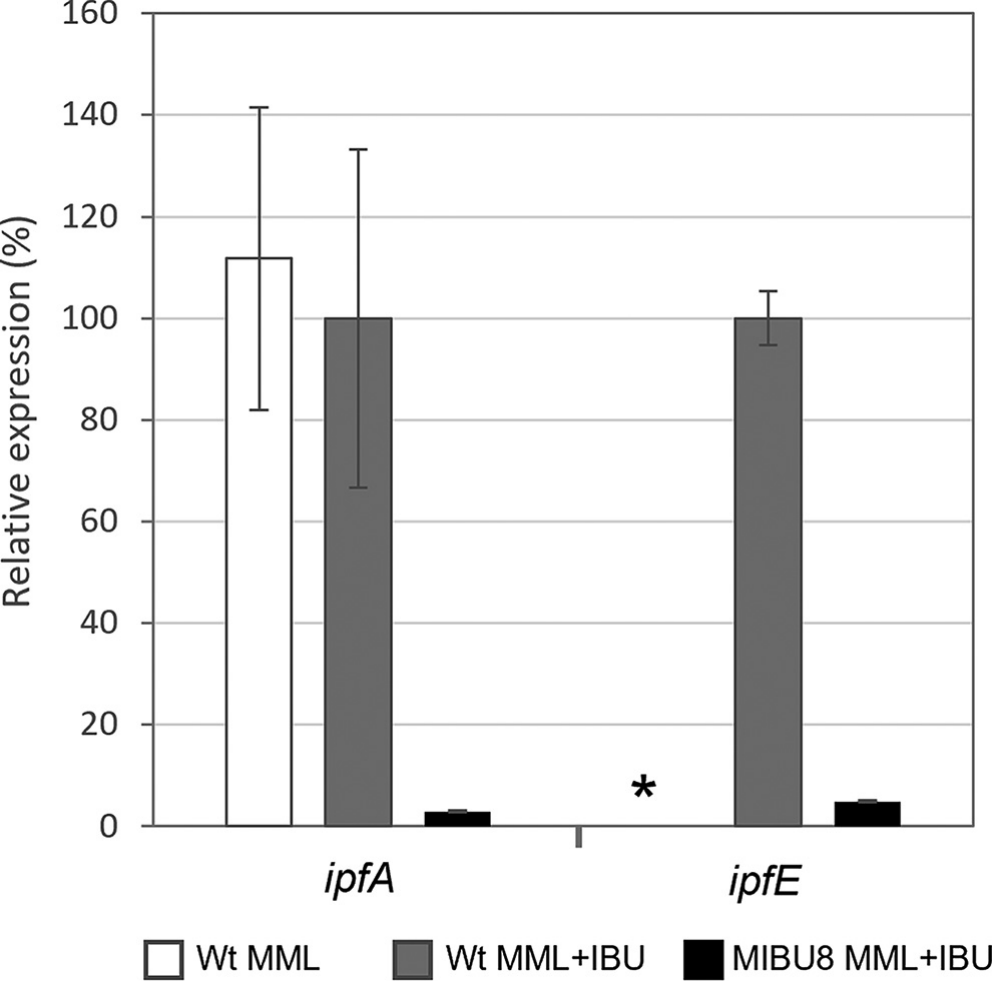
Relative expression of *ipfA* and *ipfF* genes in the MIBU8 mutant compared to MPO218. qRT-PCR analysis of the expression levels of *ipfA* and *ipfE* in the MPO218 insertion mutant MIBU8 compared to the wild-type MPO218 strain (WT) grown in complex medium (MML) and MML supplemented with IBU (MML+IBU). White bars represent the percentage of expression of the WT MPO218 in MML versus MML plus IBU; these data were not measured for *ipfE* (indicated with an asterisk). Gray bars represent the level of expression in the WT MPO218 growing in MML plus IBU (corresponding to the maximal level of expression, 100%), and black bars correspond to the percent expression in the MIBU8 mutant compared to the WT strain in MML plus IBU. Graphic represents the mean ± SD of 3 to 4 technical replicates.

On the other hand, the MIBU12 mutant carried a miniTn*5* insertion at the MPO218_05532 locus, while MIBU8 and MIBU29 mutants carried miniTn*5* insertions at the MPO218_05528 locus (numbers 19 and 23 in Fig. 2 and Table S1 at https://rio.upo.es/xmlui/handle/10433/12901), respectively. Both loci are located in the same region III flanked by the IS*6100* elements mentioned above and correspond to *ipfD* in the case of MIBU12 (number 19 in Fig. 2 and Table S1) and to an incomplete glutathione *S*-transferase-coding gene (number 23 in Fig. 2 and Table S1) in the case of MIBU8 and MIBU29 (Fig. 2). The *ipfD* gene has been previously described as involved in IBU biodegradation by Murdoch et al. (15). However, the insertion in MIBU8 and MIBU29 mutants does not localize on any of the *ipf* genes. Analyzing the gene arrangement in region III, we speculated the possibility that the *ipf* genes cluster together with the upstream genes (numbers 24, 23, and 22, respectively, in Fig. 2 and Table S1) form an operon transcribed from a putative promoter upstream of locus MPO218_05532 (number 24). To assess this possibility, we quantified the expression of several *ipf* genes located downstream the MIBU12 insertion by real-time quantitative PCR (RT-qPCR). As shown in Fig. 3, *ipfA* expression was detected when the MPO218 strain grew in MML (complex medium; see Materials and Methods) regardless of the presence of IBU in the culture medium, which indicates that *ipfA* gene expression is not regulated by IBU. However, the *ipfA* expression was almost undetectable in the MIBU8 mutant, and the same was observed for *ipfE*, indicating a polar effect of the miniTn*5* insertion on the expression of *ipf* genes downstream of the transposon (Fig. 3).

### Characterization of the leaky Ibu^−^ mutant.

**FIG 4:**
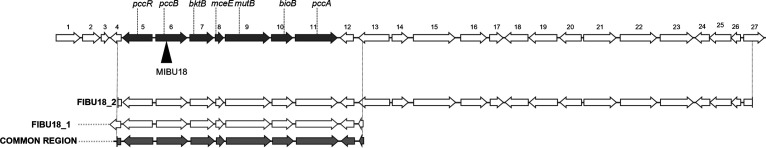
Schematic representation of the chromosomal region containing the MIBU18 insertion. The top represents the chromosomal region from locus MPO_03753 (number 1) to MPO_03779 (number 27) of the MPO218 genome (for annotation details, see Table S2 at https://rio.upo.es/xmlui/handle/10433/12901). The black triangle indicates the position of miniTn*5* in the MIBU18 mutant. Gray arrows indicate genes that form the putative *pcc* operon for metabolizing propionyl-CoA. Bottom represents the sequences present in the genomic clones FIBU18_2 and FIBU18_1 that complement the insertion mutant MIBU18 and the common DNA region (gray) of FIBU18_2 and FIBU18_1.

Analysis of the sequences flanking the miniTn*5* insertion of the leaky mutant MIBU18, which was able to produce small colonies when growing on plates with IBU as the sole carbon and energy source (Fig. S1), showed that the transposon was inserted at the locus MPO218_03758 of the MPO218 chromosome (Table S2 at https://rio.upo.es/xmlui/handle/10433/12901). According to the functional annotation of the MPO218 genome, this locus encodes a beta subunit of propionyl-CoA carboxylase carboxyl transferase (putative *pccB* gene) (Fig. 4).

To determine if this locus is actually involved in ibuprofen biodegradation, we constructed a genomic library of MPO218, containing about 20,000 independent clones with an average size of 35 kb that, according to the Clarke and Carbon formula (21), represent the entire MPO218 genome with 100% probability. We used this genomic library to complement MIBU18 and selected clones complementing the mutant phenotype. In this case, direct selection of bacteria able to grow with IBU was not possible because the mutant is actually able to grow slightly, and it was difficult to distinguish complementation just based on colony size. To circumvent this problem, we modified the standard protocol and included an enrichment step prior to selection as described in Materials and Methods. In this way, we isolated two clones (named FIBU18_1 and FIBU18_2) that complemented MIBU18 by recovering the ability to form large colonies, as the wild-type strain, when growing using IBU as the carbon source. The ends of both genomic clones were sequenced, and the sequences were compared with the MPO218 genome to determine the fragment included in both genomic clones.

The analysis of the FIBU18_2 sequence indicated that this genomic clone contains a DNA fragment of 29,833 bp, spanning from position 3917210 to position 3947093 of the MPO218 chromosome, including the *pccB* locus (Fig. 4; Table S2). This DNA fragment contains a putative operon involved in the metabolism of propionyl-CoA. On the other hand, the analysis of the sequence of FIBU18_1 showed that, at one end, the sequence corresponds to position 3928518 of the MPO218 chromosome and contains a DNA fragment, including the putative *pcc* operon (Fig. 4). Therefore, the only genomic region in common between both clones includes the putative *pcc* operon. The presence of the *pccB* gene in FIBU18_1 and FIBU18_2 was further confirmed by PCR amplification using the primers Propionyl-CoA-Fw and Propionyl-CoA-Rv (data not shown). These results confirm that the *pccB* locus is necessary for full growth on IBU.

### The dark Ibu^−^ mutants are affected in region I of pIBU218.

As mentioned above, dark mutants are mutants that are unable to grow using IBU as a carbon and energy source and that produce coloration in the presence of IBU. These mutants produce a dark brown color when grown with an alternative carbon source in the presence of IBU (Fig. S1). Like the noncolored mutants, the dark mutants were classified into two types, (i) mutants with insertions in genes related to the aromatic compound metabolism (MIBU13 and MIBU23), and (ii) other unrelated genes (MIBU1, MIBU3, and MIBU7) (Table 1).

**FIG 5:**
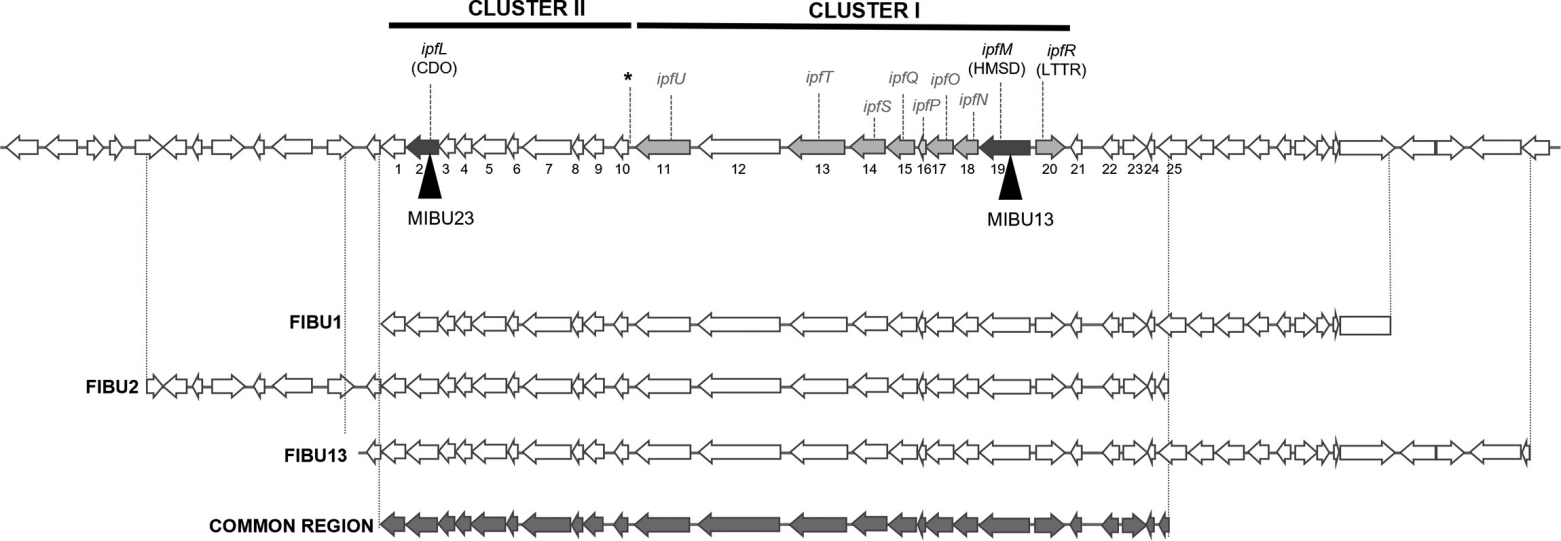
Schematic representation of the pIBU218 region affected in the dark mutants. Top represents the pIBU218 region affected in the dark insertion mutants MIBU13 and MIBU23. Arrows represent CDS, and numbers 1 to 25 indicate loci MPO218_05651 to MPO218_05475, respectively (for annotation details, see Table S3). Black triangles indicate the location of the miniTn*5* insertions in insertion mutants MIBU13 and MIBU23, and dark gray arrows indicate the genes mutated by miniTn*5*. The light gray arrows indicate other genes putatively involved in the central pathway of ibuprofen metabolism. CDO indicates the gene encoding catechol 2,3 dioxygenase, HMSD indicates the gene encoding hydroxymuconate semialdehyde dehydrogenase, and LTTR represents the gene encoding the LysR-type transcriptional regulator. The asterisk indicates the position of the possible sigma 54-dependent promoter. Bottom represents the DNA fragments included in the genomic clones of MPO218 FIBU1, FIBU2, and FIBU13 that complement both MIBU13 and MIBU23 insertion mutants. Beneath these represents the region of DNA common to all 3 fosmids (dark gray) that complement both mutants.

The analysis of the DNA sequences flanking the insertions present in MIBU13 and MIBU23 indicated that the transposons were located in the region of plasmid pIBU218 (Fig. 1) previously described as necessary for growth on IBU (16). Insertion in MIBU23 was in the MPO218_05652 locus that encodes a putative catechol 2,3-dioxygenase (CDO), while in MIBU13, it was in the MPO218_05469 locus that encodes a putative 2-hydroxymuconic semialdehyde dehydrogenase (HMSD) (numbers 2 and 19, respectively, in Fig. 5 and Table S3 at https://rio.upo.es/xmlui/handle/10433/12901).

To determine whether the phenotype of the dark mutants MIBU1, MIBU3, and MIBU7 mutants was due to the transposon insertion or rather due to plasmid instability, we assessed the presence or absence of region I and region III in these mutants by PCR amplification (Fig. 1). Results indicate that, in all cases, region III (that contains the *ipfABDEFG* cluster) was not affected. However, in none of the three mutants was there amplification of the test locus of region I, indicating that in the 3 mutants, a deletion of at least part of this region had occurred (Table 1).

Taken together, these results indicate that the phenotype of all dark mutants is related to region I of pIBU218. To assess whether mutations in this region I are directly responsible for the mutant phenotype, we complemented the MIBU1 (that contains a deletion in region I) and MIBU13 (that contains a miniTn*5* in the locus MPO218_05469; number 19 in Fig. 5 and Table S3). In this way, we identified three genomic clones, FIBU1 (with an insert of approximately 29 kb), FIBU13 (with an insert of approximately 35 kb), and FIBU 2 (with an insert of approximately 35 kb). These three clones complemented both the MIBU1 and the MIBU13 mutants, allowing the mutants to grow in minimal medium (MM) with IBU as the sole carbon and energy source and avoiding staining of the culture medium.

The three genomic clones share a DNA region that includes the genes mutated by the miniTn*5* insertions in MIBU13 and MIBU23 (Fig. 5). By comparing the sequence of the three clones, we have estimated that the pIBU218 region involved in this phenotype extends at most from the MPO218_05651 locus to the MPO218_05475 locus (numbers 1 to 25 in Fig. 5 and Table S3). We have also confirmed that FIBU13 is able to complement all the dark mutants (MIBU1, MIBU3, MIBU7, MIBU13, and MIBU23), indicating that the DNA region involved in this phenotype is the same. Together, these results indicate that all the dark mutants have a defect in the same DNA region of pIBU218 that contains the aromatic compound biodegradation genes.

**FIG 6:**
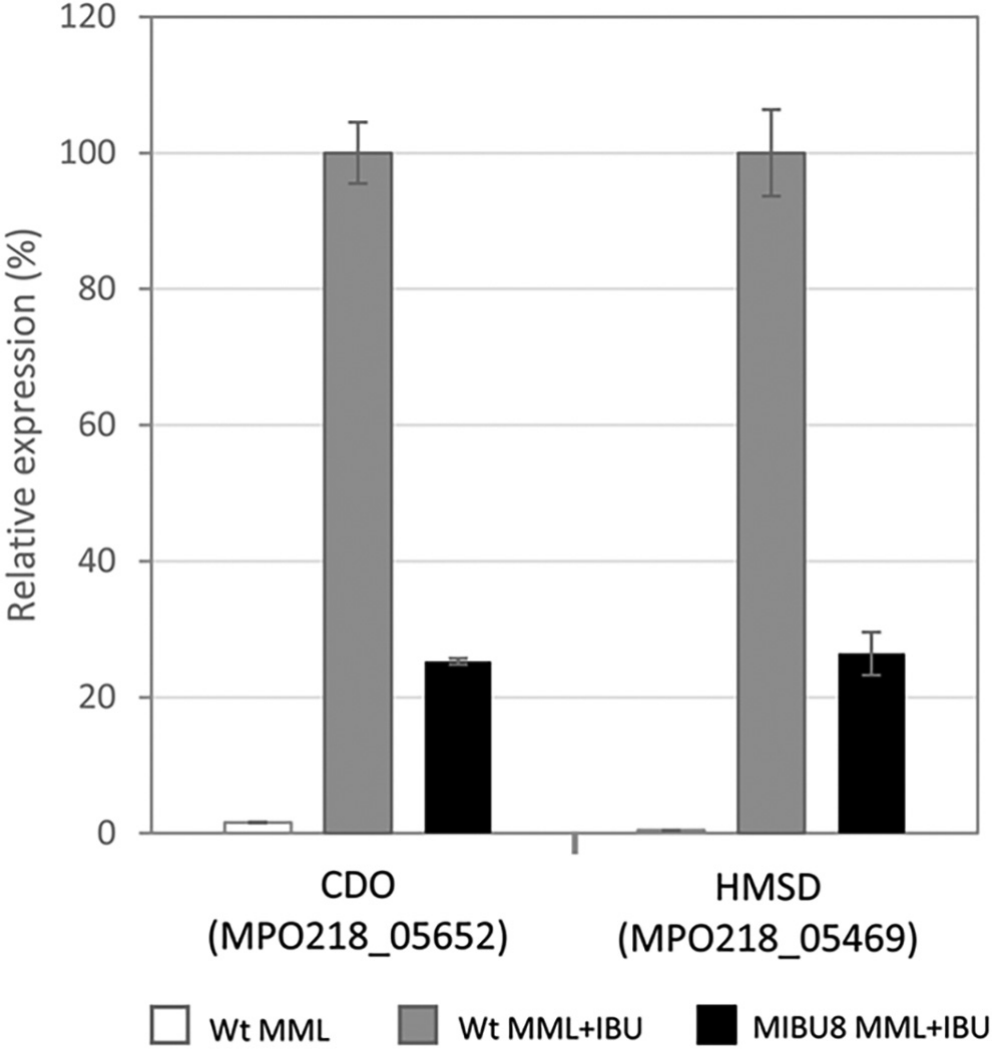
Relative expression of genes encoding CDO and HMSD. qRT-PCR analysis of the expression of genes encoding CDO (locus MPO218_05652) and HMSD (locus MPO218_05469) in the wild-type (WT) MPO218 versus the insertion mutant MIBU8 in MML in the absence and presence of IBU. The gray bars represent the expression level in MPO218 WT growing in MML plus IBU (correspond to the maximum expression level, 100%). White bar represents the percentage expression of MPO218 WT in MML compared to MML plus IBU, and the black bars correspond to the percentage of expression in the MIBU8 mutant compared to the WT strain in MML plus IBU. The graph represents the mean ± SD of 3 to 4 technical replicates.

Subsequently, we analyzed the expression of the mutated loci in MIBU23 (CDO in Fig. 5) and MIBU13 (HMSD in Fig. 5) to determine whether the presence of IBU or any metabolic intermediates of IBU degradation could affect their expression. We quantified, by RT-PCR, the expression of both loci in rich MML medium with or without IBU in the wild-type MPO218 and in the mutant MIBU8, affected in the *ipf* region required for the upper part of the IBU metabolic pathway (15) (Fig. 2). As shown in Fig. 6, transcription of both CDO and HMSD is strictly dependent on the presence of IBU in the culture medium, and induction by IBU is not repressed in rich medium. However, transcription is much lower in mutant MIBU8, thus suggesting that induction required expression of the upper pathway for IBU metabolism and, therefore, that these genes are not induced by IBU itself but by an intermediate of its biodegradation pathway.

### The dark mutants are affected in the lower part of the ibuprofen metabolic pathway.

According to Murdoch et al. (15) expression of the *ipfABDEFGHI* genes (corresponding to regions II, III, and IV in pIBU218 plasmid from our strain) without further metabolization results in the accumulation of 4-isobutylcatechol. In the presence of oxygen, this compound reacts chemically to form brown- or black-colored polycatechols, producing a dark brown coloration like what we observed in the brown mutants. According to GC-MS metabolite analysis, it has been proposed that this catecholic intermediate should subsequently be attacked by an extradiol-2,3 dioxygenase and a dehydrogenase to produce to 2-hydroxy-5-isobutylhexa-2,4-dienoic acid (19). Interestingly, MIBU23 carries an insertion in a locus that encodes a putative CDO, which is the enzymatic activity required for 4-isobutylcatechol ring opening, the next step in the metabolic pathway (Table S3 and Fig. 5).

**FIG 7:**
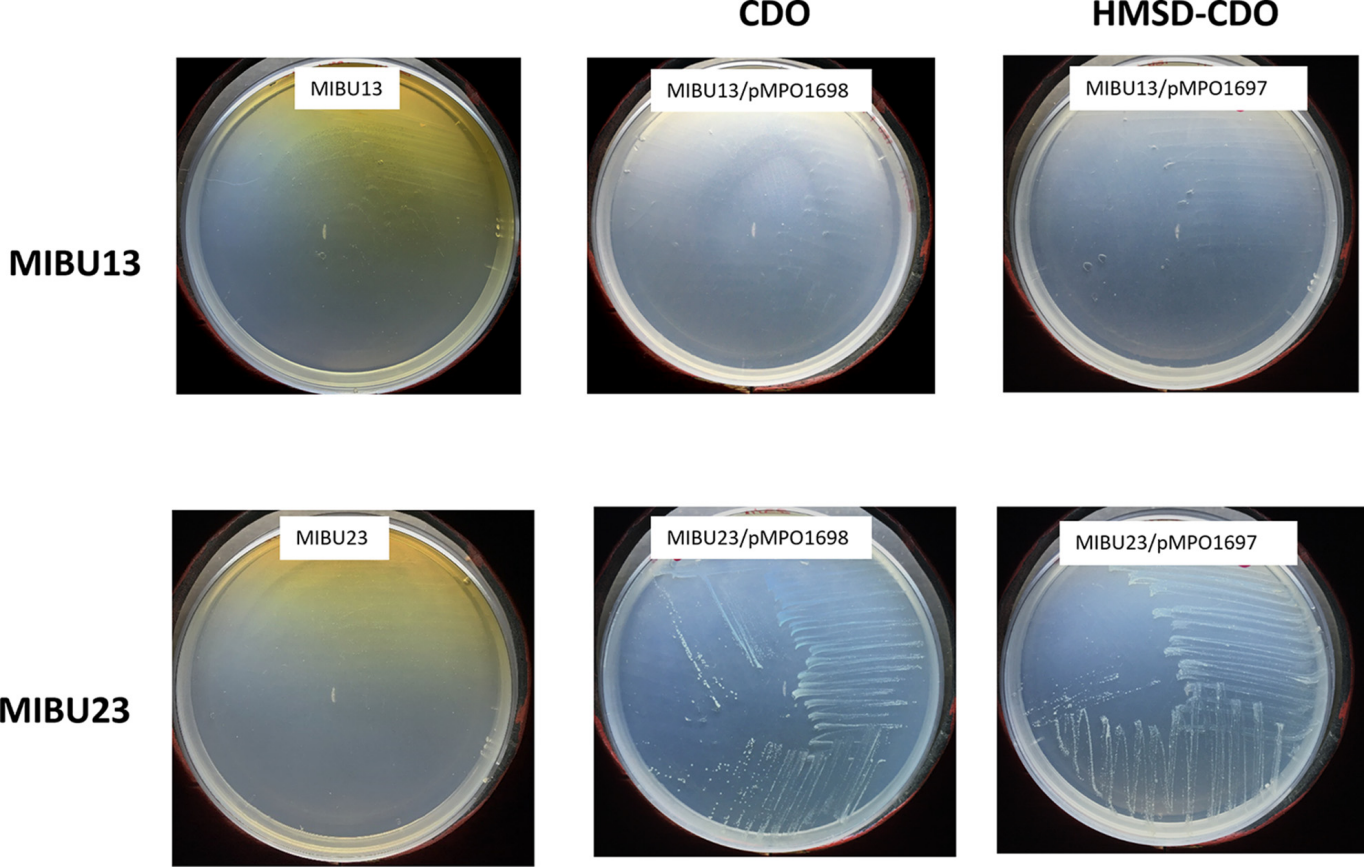
Complementation of MIBU13 and 23 mutants with plasmids pMPO1698 and pMPO1697. Images show the growth phenotype of the MPO218 insertion mutants MIBU13 and MIBU23 in MM with IBU and IPTG (left), the phenotype of both mutants complemented with the CDO activity heterologously expressed in plasmid pMPO1698 (center), and the phenotype of both mutants complemented with CDO and HMSD activities (right), heterologously expressed as an operon by plasmid pMPO1697.

**FIG 8:**
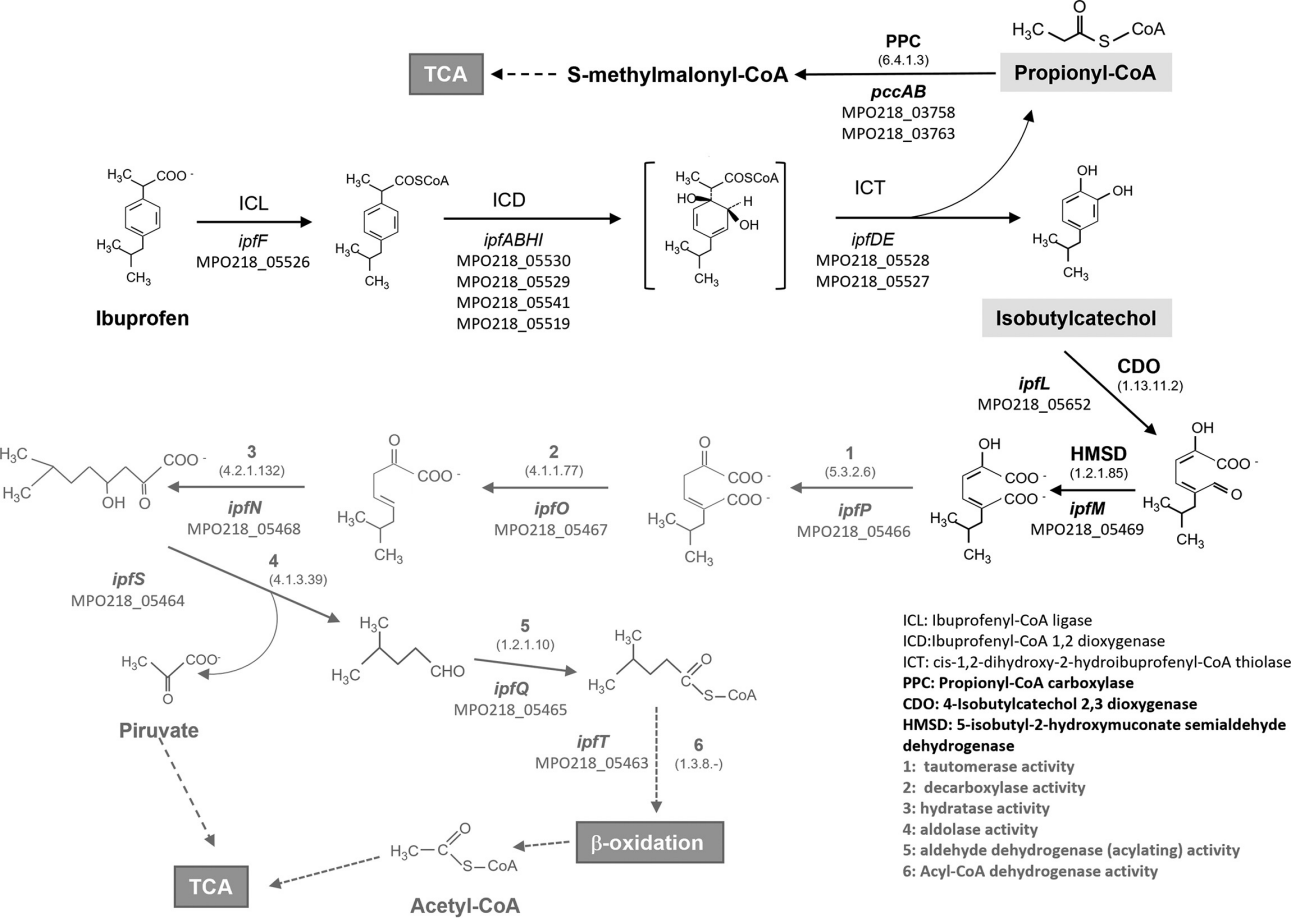
Metabolic pathway for ibuprofen degradation in R. wittichii MPO218. The steps of the upper pathway described by Murdoch et al. (15) are indicated in black and in plain text. The next steps demonstrated in this work are indicated in black and bold, and the putative steps necessary to complete the metabolic pathways and activities are shown in numbers and in gray and bold. In all cases, the assigned MPO218 loci are indicated. For the new genes described in this work, we have included EC numbers according to the protein sequence analysis.

To test if this locus encodes the enzyme required in this step, we cloned MPO218_05652 (CDO) into the vector pIZ1016 (22), generating plasmid pMPO1698, and tested whether expression of this gene was able to complement the MIBU23 defect. The results showed that expression of this gene allows MIBU23 to grow on IBU and eliminates the accumulation of the metabolic intermediate responsible for the dark phenotype (Fig. 7). This suggests that MPO218_05652 codes for the CDO necessary for the extradiolic cleavage of the 4-isobutylcatechol. Based on these results, we designated MPO218_05652 as *ipfL* (Fig. 8).

According to the proposed metabolic pathway (Fig. 8), the next step requires the action of an HMSD, which is the activity putatively encoded by the locus MPO218_05469-mutated gene in MIBU13. As before, we cloned this gene into the broad-host-range expression vector pIZ1016, generating the plasmid pMPO1696, and tested whether expression of this gene was able to complement the MIBU13 defect. The results indicated that expression of this gene does not restore the ability of MIBU13 to grow with IBU as the carbon source, and the mutant continued to accumulate dark coloration in the presence of IBU (data not shown). However, when we tested the MIBU13 mutant (HMSD) with the CDO-expressing plasmid pMPO1698, accumulation of the color of MIBU13 was eliminated, although the mutant remained unable to grow on IBU. This result suggests that the brown coloration accumulated by MIBU13 was due to lack of CDO expression in this mutant (Fig. 7).

When both genes, HMSD followed by CDO, were cloned as an artificial operon in the plasmid pMPO1697 and transferred to MIBU13, accumulation of the color disappeared. This result confirmed expression of the downstream CDO gene in the artificial operon. However, the expression of HMSD and CDO was not enough to support growth on IBU MIBU13. This suggests that, in addition to those encoding HMSD and CDO activities, other genes included in this region of pIBU218 (common region in Fig. 5) are required for MIBU13 to grow in IBU. Interestingly, analysis of this region revealed that it contains genes encoding all putative enzymatic activities necessary for the complete conversion of 2-hydroxy-5-isobutylhexa-2,4-dienoic acid to tricarboxylic acid cycle intermediates (Fig. 8).

## DISCUSSION

Although several microorganisms able to metabolize IBU have been described so far (2, 16, 17), the metabolic pathways and factors limiting biodegradation in nature remain poorly characterized. Among the bacteria described as able to grow on IBU, three different strains belonging to the family *Sphingomonadaceae* carry the same set of genes highly conserved at the DNA level, *Sphingonomas* strain ibu-2 (15), Rhizorhabdus wittichii MPO218 (16), and Sphingopyxis granuli RW412 (17).

As usual in aromatic biodegradation pathways (23), the pathway can be divided into upper pathways, which go from the aromatic compound to a central intermediate (which can be catechols or noncatecholic compounds), and the lower pathways responsible for the ring cleavage and the subsequent step to intermediates of central metabolism. Murdoch et al. (15) and, later, Aguilar-Romero et al. (17), have well described the upper part of the metabolic pathway of IBU that ends in the generation of propionyl-CoA and 4-isobutylcatechol, which should be further metabolized. In this work, we have identified the metabolic genes for the lower pathway for IBU biodegradation in MPO218 that are required to continue the metabolic pathway from the previously described intermediates.

Analysis of insertion mutants and subsequent complementation with a genomic library of MPO218 identified three different DNA regions involved in IBU metabolism that are responsible for three different mutant phenotypes. Two of the regions essential for growing on IBU are located on the pIBU218 plasmid and have been previously related to IBU metabolism (16). The third DNA region was located on the MPO218 chromosome and is not strictly necessary for growth on IBU as a carbon source, but it does enhance growth, probably by allowing full utilization of the compound.

One of these regions located on the pIBU218 plasmid is the one containing the *ipf* gene cluster described by Murdoch et al. (15). In a previous work, we demonstrated that this region is unstable in MPO218 due to the presence of different copies of an IS*6100* element (16), and deletions result in failure to grow on IBU. Here, we have demonstrated that miniTn*5* insertions mutants in this region are unable to grow on IBU and do not develop coloration of the growth medium. Although some of the mutants with a similar phenotype contained miniTn*5* insertions in locations other than the *ipf* cluster, all of them were also affected in the central *ipf* cluster (region III), which had probably been deleted by recombination between IS*6100* elements, confirming, once again, the instability of this region and the involvement of these genes in the degradation of IBU.

The second class of mutant was the leaky mutant, which was able to grow poorly on ibuprofen. This mutant was affected at a chromosomal locus annotated as *pccB* (Fig. 4 and Table S2), coding for a beta-chain subunit of propionyl-CoA carboxylase required for propionyl-CoA assimilation through the methylmalonyl-CoA pathway (24). This region also includes its hypothetical regulator (*pccR*) (25) and the other enzymatic activities necessaries for the propionyl-CoA metabolism according to KEGG pathway database (26–28) (Fig. 4 and Table S2), including *mceE*, coding a methylmalonyl-CoA epimerase; *mutB*, encoding a methylmalonyl-CoA mutase; *bioC*, involved in biosynthesis of the biotin cofactor required for the carboxylase activity; and *pccA*, coding the alpha subunit of the propionyl-CoA carboxylase. Propionyl-CoA is an intermediate in the degradation pathways of branched-chain amino acids, odd fatty acids, and branched-chain fatty acid catabolism (24, 29, 30) that can be assimilated by the methylmalonyl-CoA pathway, which is found in a wide variety of organisms (including humans) (24, 25, 31–33). Therefore, this chromosomal region of MPO218 must be involved in the metabolism of propionyl-CoA resulting from the action of the *ipfDE*-encoded thiolase (Fig. 8) (15, 17), and the mutant in this gene could not fully utilize the IBU, which could explain their lower growth than the wild type. This *pcc* region, which must be part of the central metabolism of the bacterium, is conserved in all 3 sequenced strains of R. wittichii, and a similar set of genes is also found in S. granuli RW412 and other *Sphingomonadaceae* (Fig. S2 at https://rio.upo.es/xmlui/handle/10433/12901).

The third class of mutants showed a dark phenotype when grown in the presence of IBU. All of these mutants were affected in region I of pIBU218. Our results suggest that the DNA sequence spanning from the MPO218_05651 locus to the MPO218_05475 locus (Fig. 5 and 8 and Table S3) could contain all the genes required for the lower pathway of IBU biodegradation, grouped into two clusters.

Cluster II includes genes encoding activities related to the biodegradation of phenolic compounds, including a multicomponent phenol hydrolase (numbers 6 to 9 in Fig. 5 and Table S3), a ferredoxin (number 3 in Fig. 5 and Table S3), and a catechol 2,3-dioxygenase (CDO) (number 2 in Fig. 5 and Table S3). While the first genes in cluster II should not be involved in IBU biodegradation, a mutation in the gene encoding CDO (MIBU23) results in the loss of the ability of use of IBU and the accumulation of a metabolic intermediate that produces a dark brown coloration in the presence of IBU. According to Murdoch et al. (15), the expression of the *ipfABDEFGHI* genes (the upper part of the metabolic pathway) in Escherichia coli, without further metabolization, causes the accumulation of 4-isobutylcatechol, resulting in a dark brown coloration. Thus, our results correlate the accumulation of this intermediary with the lack of CDO activity.

*In silico* analysis of the CDO amino acid sequence (mutated in MIBU23) using protein modeling and functional and folding databases indicates that it belongs to the type I extradiol dioxygenases, which is included in the vicinal oxygen chelate (VOC) superfamily (34). Besides the identified CDO in MIBU23, there are four predicted open reading frames (ORFs) that correspond to different catechol 2,3-dioxigenases on the MPO218 genome, two located on the chromosome (MPO218_01615 and MPO218_04914), one on the pUPO218 plasmid (MPO218_05676), and one on the pIBU218 plasmid (MPO218_05516). Anyway, the observation that the insertion mutant, such as MIBU23, is incapable of growing using IBU, and that this defect is complemented when the gene encoding the hypothetical CDO is expressed heterologously, indicates that this locus actually encodes the CDO activity required for the metacleavage of the catecholic intermediary generated during biodegradation of IBU, the first step in the lower pathway (Fig. 8). Therefore, we named this locus *ipfL*.

According to the metabolite analysis performed by Murdoch et al. (19), in *Sphingomonas* ibu-2, the product generated after metacleavage is further modified by an HMSD that we propose to be encoded by *ipfM* in MPO218 (Fig. 8). After a deeper sequence analysis, we have redefined the length of the MPO218_05469 ORF, situating the initial methionine at position 14 with respect to the original proposed sequence (16). The sequence analysis confirmed that this locus showed that the complete structure corresponds to the semialdehyde dehydrogenase family (35). The MIBU13 mutant contains a miniTn*5* insertion at this locus (HMSD), and, as a result, this mutant is unable to grow using IBU and shows a dark coloration. Interestingly, complementation of MIBU13 with *ipfL* (CDO) eliminates the dark coloration but not the inability to metabolize IBU. This result suggests that in MIBU13, isobutylcatechol is accumulated due to a CDO deficiency that could be due to low *ipfL* expression. However, this gene is far from *ipfM* and could hardly be part of the same operon. Interestingly, the last gene of the cluster I encodes a XylR/DmpR-type transcriptional regulator (number 11 in Fig. 5 and Table S3). Such regulators, belonging to the NtrC family, have been found to activate the expression of operons with sigma 54-dependent promoters involved in phenol degradation in many proteobacteria in the presence of inducers that are substrates of the pathway (36, 37). Therefore, it is possible that this regulator, encoded in cluster I, was required for the expression of cluster II and that the miniTn*5* insertion into *ipfM* has a polar effect on the genes downstream of the insertion, resulting in a lack of expression of the XylR/DmpR activator and, consequently, a lack of activation of cluster II. Although further experiments are required to confirm this hypothesis, visual inspection of the DNA region upstream of cluster II showed a DNA sequence that matches the consensus of the sigma 54 promoter (5′-TGGCACagcTTGCT-3′) (38), supporting this possibility.

Interestingly, complementation of MIBU13 with an artificial operon that heterologously expresses the two required activities HMSD (*ipfM*) and CDO (*ipfL*) also fails to restore the capacity of MIBU13 to grow using IBU. This mutant carries the miniTn*5* insertion in the first gene of the putative operon (Fig. 5), suggesting that, in addition to the XylR/DmpR regulator required for cluster II expression, other genes downstream of *ipfM* genes are required for growth on IBU. Interestingly, cluster I includes the following genes putatively required for further metabolism of the product generated after HMSD action (*ipfM*) via the three-step oxalocrotonate-like pathway (37) (Fig. 5 and 8 and Table S3): (i) a tautomerase (*ipfP*), (ii) a decarboxylase (*ipfO*), (iii) a hydratase (*ipfN*), (iv) an aldolase (*ipfS*), (v) an aldehyde dehydrogenase (acylating) (*ipfQ*), and (vi) an acyl-CoA dehydrogenase (*ipfT*). The final products could be further metabolized by beta-oxidation (39) and tricarboxylic acid (TCA) cycle (Fig. 8). HMSD and the other enzymatic activities required for an oxalocrotonate-like pathway are also encoded by genes that are located within the *ipf* region described previously (15, 16) (Fig. 1, region IV, and Table S1, numbers 35 to 40). However, the fact that the MIBU23 (CDO) and MIBU13 (HMSD) mutants are unable to grow on ibuprofen and that this defect is complemented with genomic clones that specifically contain clusters I and II included in region I of pIBU218 clearly indicates that these region IV genes are not involved in growth on the IBU of strain MPO218.

While the expression of *ipf* genes involved in the upper pathway of IBU biodegradation appears to be constitutive, the expression of the two clusters carrying genes involved in the lower pathway requires the presence of a metabolic intermediate of IBU biodegradation. This conclusion can be inferred from the results of the *ipfL* (CDO) and *ipfM* (HMSD) gene expression genes in the MIBU8 mutant (Fig. 6). Interestingly, the first locus upstream of cluster I and in the opposite direction (number 20 in Fig. 5) encodes a hypothetical ClcR regulator that belongs to the LysR-type regulators. LysR-type regulators are involved in the regulation of a variety of degradation pathways, including catechol metacleavage pathways (36). In general, the gene for this type of regulator is divergently located upstream of its target-regulated operon, and the regulator activates transcription in response to an inducer, usually a pathway intermediate. All these features are consistent with the genetic organization and expression pattern observed for cluster I, supporting the hypothesis that the genes required for the lower pathway of IBU metabolism are regulated by two different regulatory factors.

Based on our results, we propose a putative complete pathway for IBU biodegradation in R. wittichii MPO218 (Fig. 8). In addition to the *ipf* genes previously described for the upper IBU biodegradation pathway (regions II, III, and IV in pIBU218), our results indicate that clusters I and II (region I in pIBU218) contain the genes required for the lower IBU biodegradation pathway. The fact that most of the genes encoded by the cluster II are not involved in IBU biodegradation and that this cluster contains a phenol monooxygenase that should not be involved in IBU metabolism suggests that clusters I and II could have been originally involved in the metabolism of a different aromatic compound and could have been recruited for IBU metabolism in MPO218. The expression of both clusters seems to be coordinately regulated and induced by an aromatic metabolic intermediate of IBU degradation, which suggests that not only the catabolic genes but also the regulatory genes have evolved to recognize IBU biodegradation metabolites (40). Further analysis is required to determine the nature of the metabolic intermediate and the precise regulation of both clusters. Identification of the genes required for biodegradation, as well as their regulation, is important to understand and predict the behavior of these bacteria in complex environments such as wastewater treatment plants and to maximize their potential for contaminant removal and design-optimized biocatalysts for the degradation of drugs and other emerging contaminants.

## MATERIALS AND METHODS

### Bacterial strains and growth conditions.

Luria-Bertani (LB) medium or LB agar was used as the standard growth medium for E. coli. Bacteria were aerobically grown at 37°C. MPO218 was aerobically grown in the rich medium MML or minimal medium (MM) supplemented with different carbon sources (41) at 30°C. IBU 4 mM or β-hydroxybutyrate (BHB) 14 mM was used as the carbon source. Antibiotics were used at the following concentrations: 10 mg L^−1^ tetracycline, 100 mg L^−1^ streptomycin, 10 mg L^−1^ ampicillin, 25 mg L^−1^ kanamycin, and 7.5 mg L^−1^ gentamicin.

### Molecular biology general procedures.

DNA manipulations were performed according to standard procedures (42). All oligonucleotides used in this study are described in Table 2. For colony PCR, colonies were resuspended in 50 μL of water, boiled for 5 min, and centrifuged for 30 min at 13,000 rpm, and 2 μL of supernatant was added to the PCR assay. For PCR amplification, Illustra PuReTaq ready-to-go PCR beads kit (GE Healthcare) or Q5 high-fidelity polymerase (New England Biolabs) was used. For purification of DNA fragment, Illustra GFX PCR DNA and gel band purification kit (GE Healthcare) were used. DNA sequencing was performed by Secugen SL, and DNA sequences were analyzed by BLAST using the NCBI web service (https://blast.ncbi.nlm.nih.gov/Blast.cgi).

**TABLE 2:** Oligonucleotide PCR primers

Primer name	Sequence (5′–3′)	Target
*rrn*-f27	AGAGTTTGATCMTGGCTCAG	16s RNA gene
*1406R*	ACGGGCGGTGTGT(AC)C	16s RNA gene
CEKG-2A	GGCCACGCGTCGACTAGTACNNNNNNNNNNAGAG	miniTn*5*-Km
CEKG-2B	GGCCACGCGTCGACTAGTACNNNNNNNNNNACGCC	miniTn*5*-Km
CEKG-2C	GGCCACGCGTCGACTAGTACNNNNNNNNNNGATAT	miniTn*5*-Km
CEKG-4	GGCCACGCGTCGACTAGTAC	miniTn*5*-Km
mTn5mapIR	AGCTTGCTCAATCAATCAC	miniTn*5*-Km
mTn5mapIIR	CACCTCAATCAATCACCGGATC	miniTn*5*-Km
Propionyl-CoA-Fw	TTCGACAACGACATCGAGGC	MPO218_03758
Propionyl-CoA-Rv	ACGAGTTGATGTCGAGACAG	MPO218_03758
IpfG int-fw	GTCCCTTTCGTGATCCAGAC	MPO218_05524
ipfG-Int-Rv	AGGTCAAGAATGGTCGATGG	MPO218_05524
q-ipfE-Fw	CCGCGGGGCTGTTCTCTC	MPO218_05527
q-ipfE-Rv	TCGGCCAGCTCGATCAATC	MPO218_05527
ipfA-Fw	AACAAGCTGACCCACACAGG	MPO218_05530
ipfA-Rv	CGATGCGCGCAACGGTTGG	MPO218_05530
q-ipfA-Fw	GGCAAGTACACAAACGATCAC	MPO218_05530
q-ipfA-Rv	TACGGTCTCGCTCTATCAAG	MPO218_05530
BphC-Fw	TCGCTGCATAGAACGACACC	MPO218_05533
BphC-Rv	TGATGGAAGCAGGCGTCACC	MPO218_05533
CDO-SpeI-Fw	TAACTAGTAAGACCGAACCCAAGCG	MPO218_05652
2-CDO-SacI-Rv	TTGAGCTCGTTCGCGCATGGTCAGC	MPO218_05652
q-CDO-Fw	TGTGCGGGTTCTCGATATCG	MPO218_05652
q-CDO-Rv	CGCCTTGTAGTAGGTTTTGC	MPO218_05652
2_HMDH-HindIII-Fw	ATAAGCTTACCCGCTACTATGGATGC	MPO218_05469
HMDH-BamHI-Rv	TTGGATCCAACCGTCGACGATGTCC	MPO218_05469
q-HMDH-Fw	CGGAGATCCGGCATTTCATC	MPO218_05469
q-HMDH-Rv	ACGCCCACCCTCGTTCAC	MPO218_05469
M13-Fw	GTAAAACGACGGCCAG	pMOP1669
M13-Rv	CAGGAAACAGCTATGA	pMOP1669
SalI-end amp	TTATGTCGACGGGATTTTGGTCATGAG	pBlueScript II SK (+)
stuI-amp inicio	TTATAGGCCTTTACAATTTAGGTGGCAC	pBlueScript II SK (+)

### Cloning and expression of the genes encoding de CDO and the HMSD activities.

The MPO218_05469 locus encoding the putative HMSD activity was PCR amplified from MPO218 genomic DNA using primers 2_HMDH-HindIII-Fw and HMDH-BamHI-Rv. The PCR product was digested at one end with HindIII and cloned into pIZ1016 digested with HindIII-SmaI, generating plasmid pMPO1696. The MPO218_05652 locus encoding the putative CDO activity was PCR amplified from MPO218 genomic DNA using primers CDO-SpeI-Fw and CDO-SacI-Rv. The PCR product was digested with SpeI-SacI and cloned into pIZ1016 digested with XbaI, filled with Klenow, and subsequently digested with SpeI to generate plasmid pMPO1698. Alternatively, the same fragment was cloned into pMPO1696 and digested as before (XbaI-Klenow-SpeI) to generate plasmid pMPO1697, which contains an artificial operon with the locus encoding HMSD upstream of the locus encoding CDO.

Plasmids pMPO1696, pMPO1697, and pMPO1698 were electroporated into the appropriate strains, and gene expression was induced with IPTG (isopropyl-β-d-thiogalactopyranoside) (1 mM) supplemented on media plates.

### Mutagenesis with miniTn*5*-Km.

Mutagenesis with the miniTn*5* transposon was carried out by triparental matings (43), using DH5α λpir/pUT-miniTn*5*-Km as donor, MPO218 as recipient, and DH5α/pRK2013 as the auxiliary strain. Overnight cultures were diluted to an *A*_600_ of 0.1 and incubated to an *A*_600_ of approximately 0.5. Next, 15 mL of the MPO218 culture and 3 mL of the donor and auxiliary strain cultures were harvested by centrifugation, washed, and resuspended in phosphate buffer (PB) 1×. The 3 strains were mixed and centrifuged for 10 min at 13,000 rpm, and the pellet was resuspended in 50 μL of 1× BP and placed dropwise on an MML plate and incubated at 30°C overnight. The mating mixtures were then diluted and plated onto 30 MML plates supplemented with kanamycin (Km) and streptomycin (Str) and incubated at 30°C for 10 days. From the 30 plates, 12,000 independent colonies were randomly picked and streaked on both IBU-supplemented MM and BHB-supplemented MM plates and incubated at 30°C for 5 days. After this time, colonies that grew on BHB but not on IBU were selected. These colonies were subsequently segregated on MM and MML plates supplemented with IBU, and the different mutants were classified according to the difference in growth with the wild type and the color they produced in the culture medium.

### Mapping of miniTn*5*-Km insertions by PCR with arbitrary primers.

To determine the miniTn*5* insertion sites, the flanking regions of the transposon insertions were amplified following the sequential PCR protocol described by López-Sánchez et al. (44). The PCR products were purified and sequenced using mapTn5IIr as the primer, and the sequences obtained were located in the MPO218 genome by BLAST analyses. Once insertions were mapped, their location was confirmed by PCR amplification with specific oligonucleotides.

### MPO218 genomic library construction.

The pJC8 vector (45) was modified to obtain plasmid pMPO1669. First, the fragment corresponding to the Tc resistance gene was removed by digestion with StuI and SalI enzymes and replaced with the Ap resistance gene with its promoter, obtained by PCR from the pBlueScript II SK (+) vector by amplification with StuI-amp-initial and SalI-end-amp oligonucleotides. The resulting plasmid was designated pMPO1668. Subsequently, pMPO1668 was digested with BamHI and ligated to remove the fragment conferring resistance to gentamicin (Gm), thus yielding the plasmid pMPO1669.

For the construction of the MPO218 genomic library, the protocol described by Terrón-González et al. was followed with some variations (46). MPO218 genomic DNA was extracted, following the instructions of the Wizard genomic DNA purification kit (Promega), starting from 5 mL of 10 saturated cultures in MML. Next, end repair was performed following the instructions of the end-repair enzyme mix kit (Epicenter) in 6 independent reactions with approximately 5 μg of DNA per reaction. After electrophoresis on a 0.7% (wt/vol) low-melting-point agarose gel at 4°C, for 13 h at 60 V, the bands with DNA of suitable size (30 to 40 kb) were purified using GELase kit (Epicentre). The DNA was then concentrated using Amicon Ultra 100K filters (Millipore).

The pMPO1669 vector was linearized by the PmlI enzyme (New England Biolabs; isoschizomer of Eco72I), dephosphorylated with shrimp alkaline phosphatase (USB), and concentrated using Amicon Ultra 100K membranes (Millipore). After ligation, the genomic library was packaged using MaxPlax Lambda packaging extracts (Epicenter) and transducted to EPI300-T1R.

The constructed library, consisting of about 20,000 independent clones, was mass stored in a single EPI300-T1 culture. Restriction analyses of randomly selected clones indicated that each of the clones contained different DNA inserts with an average size of 35 kb; thus, the library harbored approximately 700 Mb of genomic DNA, more than 100-fold the length of the MPO218 genome (6 Mb).

### Complementation of miniTn*5*-Km insertion mutants with the MPO218 genomic library.

Complementation of miniTn*5*-Km (recipient strains) with the MPO218 genomic library (donor) was carried out by triparental mating as explained above. After incubation at 30°C overnight, the mating mixtures were diluted and plated on MM with IBU 4 mM to select the mutants complemented by the genomic library.

In the case of the MIBU18 mutant, the protocol was varied. In this case, the mating mixture was resuspended in 20 mL of MM plus IBU (4 mM) and incubated for 7 days at 30°C together with a control culture of recipient MIBU18. After this incubation period, 2 enrichment cultures were carried out, which consisted of 5 mL of the previously enriched culture mixed with 15 mL of MM plus IBU (4 mM) with a 7-day incubation period. When a difference in growth was observed by comparing to the control culture, serial dilutions were plated on MM with IBU (4 mM) and MM, IBU, kanamycin at 25 μg/mL (Km25), and ampicillin at 100 μg/mL (Ap100) plates and incubated for 5 days at 30°C.

### RT-qPCR assays.

Cells were grown in MML supplemented with or without IBU until *A*_600_ of 0.8 was reached, and RNA extraction was carried out as described before (47). After DNase I treatment with a DNA-free kit (Ambion), the RNA was purified using RNAeasy columns (Qiagen). RNA quality was confirmed by agarose gel electrophoresis. The absence of contaminating DNA was confirmed by PCR amplification of the 16S rRNA gene with primers f27 and r519.

Prior to cDNA synthesis, equivalent amounts of RNA from three biological replicates of each sample were mixed and used in the expression analysis. RNA retrotranscription was carried out using the high-capacity cDNA reverse transcription kit (Thermo Fisher Scientific) according to the protocol provided by the manufacturer. After retrotranscription, the samples were cleaned up using the QIAquick PCR purification kit (Qiagen). The resulting cDNA was amplified using 0.3 mM of each primer (Table 2) as previously described (48). The results are the average of 3 technical replicates.

### Sequence analysis.

The structural annotation of the MPO218 genome was performed in a previous work (16) using Prokka (49), and the predicted proteins were functionally annotated using Sma3S v2 and UniProt Bacteria (50). These annotations were used to characterize both genes and clusters. Gene ontology (GO) terms were used to do the functional enrichment, using the topGO library in R programming language. All genes relevant to the present work have been further manually annotated using BLASTp and BLAST conserved domain at NCBI. Protein modeling and folding and functional predictions of the CDO and HMSD were carried out using Swiss-Model server (51), Phyre2 web portal (52), KEGG database resource (26–28), and UniProt database (53).

### Data availability.

The MPO218 chromosome and plasmid pIBU218 sequences are available in GenBank under accession numbers NZ_CP059319 and NZ_CP059320, respectively. The MPO218 annotation available from NCBI differs slightly from our annotation at some loci. To facilitate sequence analysis, we have included additional information on locus tag matching in the supplemental material(Supplementary tables and supplementary FASTA sequences, https://rio.upo.es/xmlui/handle/10433/12901). In addition, we have included the DNA sequences analyzed in this work as supplemental information.
